# Supplementary material for: Empirical methods for controlling false positives and estimating confidence in ChIP-Seq peaks
Source: BMC Bioinformatics. 2008 Dec 5;9:523. doi: 10.1186/1471-2105-9-523 (PMC2628906; doi:10.1186/1471-2105-9-523)
Supplement: Additional File 1 — A variety of html documents from the USeq web site detailing the available applications, their best usage, output file type descriptions, command line menus, etc. [file 1471-2105-9-523-S1.zip › USeqUserGuides/JavaDoc.zip!/JavaDoc/trans/misc/Wig2Bar.html]

Wig2Bar


|  |  |  |
| --- | --- | --- |
| **Overview** \| **Package** \| **Class** \| **Use** \| **Tree** \| **Deprecated** \| **Index** \| **Help** | |  |
| **PREV CLASS**   **NEXT CLASS** | **FRAMES**    **NO FRAMES**     **All Classes** |  |
| SUMMARY: NESTED \| FIELD \| CONSTR \| METHOD | DETAIL: FIELD \| CONSTR \| METHOD |  |


---


## trans.misc Class Wig2Bar

```
java.lang.Object
  trans.misc.Wig2Bar
```

---

``` public class Wig2Bar extends java.lang.Object ```

---

**Constructor Summary**

`Wig2Bar(java.lang.String[] args)`


| **Method Summary** | |
| --- | --- |
| `static void` | `main(java.lang.String[] args)` |
| `static GrGraph[]` | `parseFixedStepWigFile(java.io.File wigFile, float skipValue)`             Parses a fixedStep wig file, skips any track type data. |
| `static GrGraph[]` | `parseVariableStepWigFile(java.io.File wigFile, float skipValue)`             Parses a variableStep wig file, skips any track type data. |
| `static java.lang.String` | `parseWigFileType(java.io.File wigFile)`             Looks for the first line that begins with 'fixedStep' or 'variableStep', if none found returns null, otherwise returns one of those two names. |
| `static void` | `printDocs()` |
| `void` | `processArgs(java.lang.String[] args)`             This method will process each argument and assign new varibles |

**Methods inherited from class java.lang.Object**

`equals, getClass, hashCode, notify, notifyAll, toString, wait, wait, wait`

**Constructor Detail**

### Wig2Bar

```
public Wig2Bar(java.lang.String[] args)
```


**Method Detail**

### parseWigFileType

```
public static java.lang.String parseWigFileType(java.io.File wigFile)
                                         throws java.lang.Exception
```

:   Looks for the first line that begins with 'fixedStep' or 'variableStep', if none found returns null, otherwise returns one of those
    two names.

    :   **Throws:**: `java.lang.Exception`

---


### parseVariableStepWigFile

```
public static GrGraph[] parseVariableStepWigFile(java.io.File wigFile,
                                                 float skipValue)
                                          throws java.lang.Exception
```

:   Parses a variableStep wig file, skips any track type data. Span is assumed to be 1.
    Should look something like:
    variableStep chrom=chr19 span=1
    59304701 10.0
    59304901 12.5
    59305401 15.0
    59305601 17.5
    Subtracts 1 from each position to convert from 1-relative coordinates specified from UCSC Wig fromat
    to interbase coordinates.
    Skips lines with designated skipValue

    :   **Throws:**: `java.lang.Exception`

---


### parseFixedStepWigFile

```
public static GrGraph[] parseFixedStepWigFile(java.io.File wigFile,
                                              float skipValue)
                                       throws java.lang.Exception
```

:   Parses a fixedStep wig file, skips any track type data.
    Should look something like:
    fixedStep chrom=chrY start=668 step=1
    0.012
    0.021
    0.028
    0.033
    0.036
    Subtracts 1 from each position to convert from 1-relative coordinates specified from UCSC Wig format
    to interbase coordinates.

    :   **Throws:**: `java.lang.Exception`

---


### main

```
public static void main(java.lang.String[] args)
```

---


### processArgs

```
public void processArgs(java.lang.String[] args)
```

:   This method will process each argument and assign new varibles

---


### printDocs

```
public static void printDocs()
```


---


|  |  |  |
| --- | --- | --- |
| **Overview** \| **Package** \| **Class** \| **Use** \| **Tree** \| **Deprecated** \| **Index** \| **Help** | |  |
| **PREV CLASS**   **NEXT CLASS** | **FRAMES**    **NO FRAMES**     **All Classes** |  |
| SUMMARY: NESTED \| FIELD \| CONSTR \| METHOD | DETAIL: FIELD \| CONSTR \| METHOD |  |


---
